# Heart Rate Recovery (HRR) Is Not a Singular Predictor for Physical Fitness

**DOI:** 10.3390/ijerph20010792

**Published:** 2022-12-31

**Authors:** Claudia Römer, Bernd Wolfarth

**Affiliations:** Department of Sports Medicine, Charité-Universitätsmedizin Berlin, Corporate Member of Freie Universität Berlin, Humboldt-Universität zu Berlin, 10117 Berlin, Germany

**Keywords:** heart rate recovery, physical fitness, preventive medicine, public health

## Abstract

Background: For optimal recommendations in cardiovascular training for the general population, knowing the essential parameters for physical fitness is required. Heart rate recovery (HRR) is an easy-to-measure parameter and is discussed to derive the physical fitness of an individual subject. This study evaluates HRR as a potential physical fitness parameter for public health programs, as it is measured in every ergometry. Methods: In this retrospective cross-sectional study, we analyzed HRR regarding physical fitness (W/kg (IAT: individual anaerobic threshold)). In total, we analyzed 1234 performance protocols in cycle ergometry. Significance tests (*p* < 0.001) and multiple linear regression were performed. Results: The analysis of HRR and weight-related performance showed a significant correlation with a moderate coefficient of determination (R^2^ = 0.250). The coefficient of determination increases from very weak correlation levels at 1 min post-workout towards weak to moderate levels of correlation at 5 min post-workout. Conclusions: In this study HRR and the weight-related performance at the IAT showed a significant correlation with a mean strength. Thus, a prediction or conclusion on physical performance based singularly on HRR decrease is not recommended. However, in preventive medicine, HRR should be measured and observed on a long-term basis, for analysis of vagal activity and to draw to inferences of mortality.

## 1. Introduction

Numerous studies have shown an increased risk of cardiovascular diseases with physical inactivity [1]. The consequences of cardiovascular diseases such as diabetes mellitus, arterial hypertension and obesity will in future result in an increasing burden of disease for our society and the financing of the health system [2,3].

At the same time, it has been proven that regular physical activity reduces morbidity and mortality [3,4]. Numerous retrospective examinations as well as prospective intervention analyses showed the advantage of sporting activity for prevention of cardiovascular diseases. Furthermore, it could also be shown that sporting activity lowers the risk for cardiovascular diseases, lung diseases, chronic inflammatory diseases and tumor diseases [5,6,7,8,9].

For an optimal training recommendation an accurate knowledge of the individual’s physical fitness is necessary [10]. One of the most elaborate methods to determine physical fitness is a lactate performance diagnostic test [10]. The determination of individual fitness and parameters at the individual anaerobic threshold (IAT) is cost- and time-consuming and remains mainly reserved for competitive athletes and ambitious recreational athletes [11]. To prevent cardiovascular diseases, it is necessary to establish simple methods of performance measurement for patients and sports beginners. A simple method for determining individual physical performance could be the observation of the heart rate recovery (HRR). Examinations in athletes have shown a correlation between a faster HRR with a better physical fitness [12,13,14]. In contrast, Daanen et al. showed a negative correlation of physical performance and HRR in a systematic review [15]. This study included five cross-sectional studies and eight longitudinal studies. Three out of five cross studies showed a faster decline of HRR. The investigated longitudinal studies predominantly showed a connection of HRR and the respective training state of the subjects [15]. It could be shown that HR decreased faster in well-trained subjects, remained constant with no change in the training plan and decreased slower with a reduction in the amount of exercise [15]. Daanen et al. concluded that HRR is an essential parameter for training control, however, it is also dependent on many other factors [15]. However, all included studies showed small study populations and different study designs [15,16,17,18].

It is now to be considered whether this data can be confirmed in a larger heterogeneous data collective. The HRR data is routinely documented after every ergometry but to date remains largely unused. In this way, individual training recommendations could be derived by the supervising physician after a routine cycle ergometry.

There is a need for more research in preventive medicine to include better preventive training control for the main population [1]. This study was conducted to close the research gap on HRR recovery by analyzing a heterogenous dataset of over 1234 cycle ergometry examinations and evaluating HRR as a potential parameter for training control in the main population.

## 2. Materials and Methods

In a retrospective analysis, secondary data of the Sports Medicine Institute of the University Medical Center Charité, Berlin, and Humboldt University, Berlin, were analyzed for the correlation of HRR and individual physical performance. All ergometry protocols performed between 2015 and 2017 were derived from the institutional sports medicine information system. Patients were excluded for the following reasons: For the present analysis, patients (I) with missing lactate data (II) with missing heart rate data and (III) with insufficient protocols and implausible data.

The study was conducted in accordance with the Declaration of Helsinki and with the approval of the local ethics committee of Humboldt University, Berlin.

Continuous variables were tested for normal distribution using the Kolmogorov–Smirnov test and descriptive analysis; variables not following a normal distribution are presented as median and interquartile range (IQR) and were compared using the Mann–Whitney U-Test. Baseline subject characteristics were acquired as shown in Table 1. For individual physical performance, power-per-kilogram of body weight at the individual anaerobic threshold (IAT) was used and is abbreviated as W/kg (IAT). Examination of the existing dependency between the weight-related performance and HRR is shown in the following paragraph. First, the variables HRR-absolute (represents the absolute drop in heart rate over the post-exercise time of 5 min, in bpm) and HRR-relative (in %) were generated. The correlation analysis of the HRR-absolute and the weight-related performance W/kg (IAT) was performed subsequently. For correlation analysis, the Pearson correlation coefficient and root mean square error (RMSE) were used. A two-sided significance level of α 0.001 was defined as appropriate to indicate statistical significance. All statistical analyses were performed using the SPSS software (IBM Corp. Released 2016. IBM SPSS Statistics for Windows, Version 25.0., IBM Corp., Armonk, NY, USA).

## 3. Results

**Figure 1:**
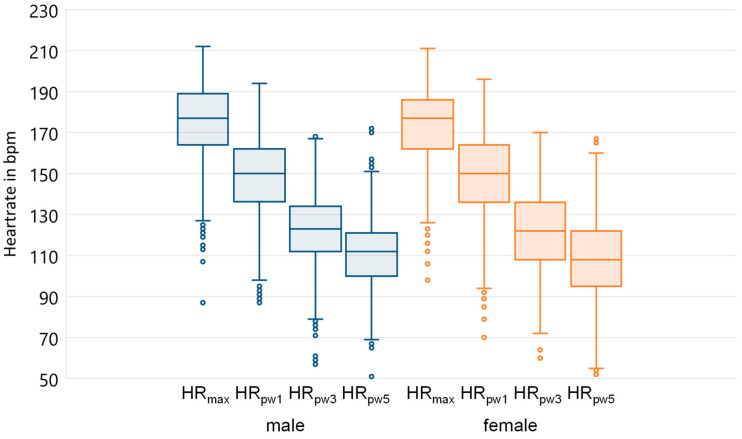
HRR after one, three and five minutes for male and female athletes.

**Figure 2:**
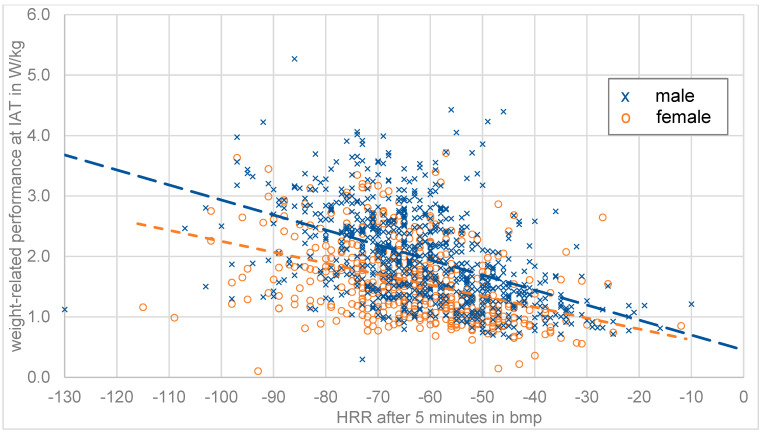
Weight-related performance depending on HRR after 5 min for all 1234 athletes in our dataset.

Figure 1 shows the maximum heart rate as well as the measured HRR presented after one, three and five minutes. Both men and women show a wide dispersion and a similar decrease in the course of the HRR curve. For further correlation analysis the variable HRR-absolute was generated. Figure 2 shows the correlation of HRR and W/kg (IAT) for all athletes.

**Figure 3:**
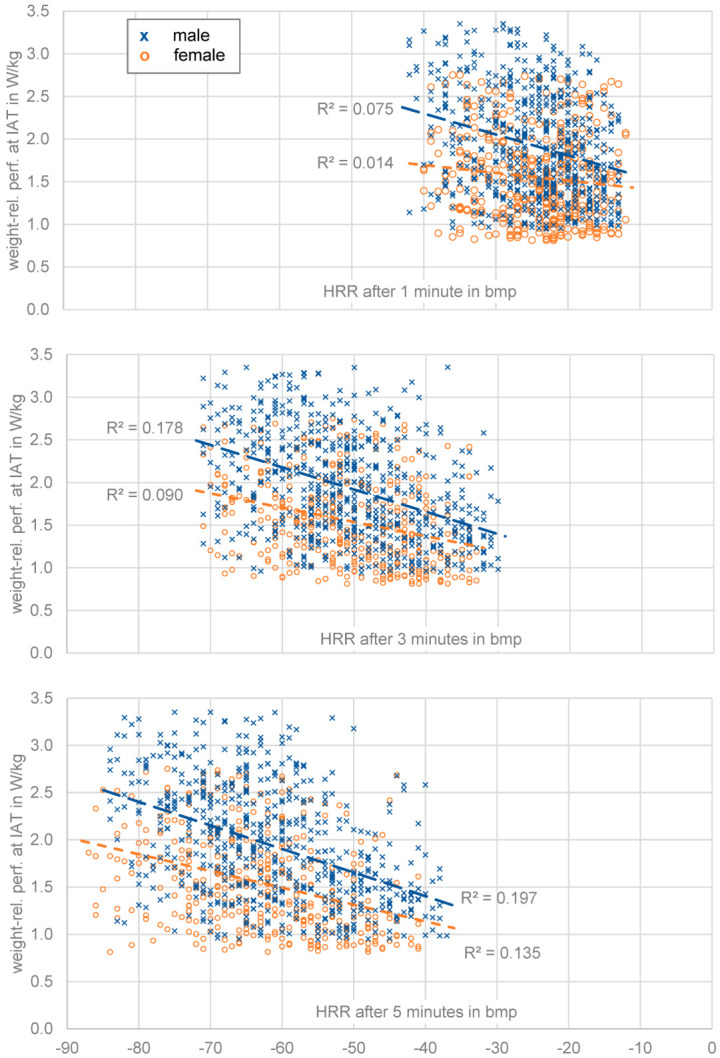
Weight-related performance depending on HRR after 1, 3, and 5 min for male and female athletes.

In Figure 2 the correlation of the weight-related performance and HRR after 5 min in the data set shows a weak negative correlation, the same is true for HRR after 1 min and 3 min. This means that the weight-related performance can only be determined with large deviations based on HRR. This observation is also apparent when gender differences are analyzed, since gender is a relevant parameter for HR and HRR in the literature. In both male and female subjects, a weak correlation between weight-related performance and HRR could be observed. However, the data shown in Figure 2 contains a number of significant outliers (i.e., weight related performance values of 0.15 W/kg or 5.3 W/kg and HRR after 5 min of 10 bpm or 130 bpm) that would greatly influence the following linear regression analysis. Thus, an outlier removal procedure for HRR values for 1, 3, and 5 min and the weight-related performance values at IAT was performed; removing values below the 5th percentile and above the 95th percentile for each individual parameter and gender. The resulting data for HRR 1, 3, and 5 min post-workout is shown in Figure 3.

Due to this outlier removal process, the number of available data points with values for both HRR and weight-related performance are reduced from 1234 athletes for

HRR after 1 min to n = 1005 (620 male, 385 female)
HRR after 3 min to n = 1009 (625 male, 384 female)
HRR after 5 min to n = 1010 (626 male, 384 female)

In Figure 3 it can be seen that for all post-workout timespans a negative correlation between weight-related performances and HRR exists for both genders. The coefficient of determination increases from very weak correlation levels (R^2^ = 0.075/0.014) at 1 min post-workout towards weak to moderate levels of correlation (R^2^ = 0.197/0.135) at 5 min post-workout. In all three cases the coefficients of determination were higher for male athletes.

The increase in R^2^ over time can be explained to some degree as the HRR at a certain time naturally includes the HRR at earlier points in time (i.e., HRR_1min_ in HRR_3min_ and in turn HRR_3min_ in HRR_5min_). Thus, the level of information by observing a longer, yet still relevant (cf. Figure 1) timespan increases. Following this reasoning, we provide the results of a multiple linear regression analysis in Table 2. There it can be seen that both HRR after 1 and 3 min do not surpass the significance level of α 0.001 if HRR after 5 min is also used as a parameter.

As discussed above, the lack of significance for HRR_1min_ and HRR_3min_ in Table 2 are due to the inherent information regarding prior HRR values in HRR_5min_. Therefore, we propose to transform the parameters used for multiple linear regression by considering only the additional heart rate recovery after the prior HRR values, thereby reducing the inherent correlation of subsequent HRR measurements. This leads to (HRR_3min_ − HRR_1min_) and (HRR_5min_ − HRR_3min_) heart rate recovery deltas, besides the existing HRR_1min_ value. The resulting multiple linear regression metrics are shown in Table 3.

As can be seen in Table 3, the parameter transformation into separate HRR deltas results in all time-ranges being highly significant, and the overall adjusted coefficient of determination increasing to R^2^ = 0.250. At the same time, considering this also includes the significant influence of gender, it must be concluded that heart rate recovery should not be used as a singular predictor for physical fitness.

## 4. Discussion

### 4.1. Heart Rate Recovery (HRR) Is Not a Singular Predictor for Physical Fitness

In this retrospective analysis, HRR was examined for prediction of physical performance. In total 1234 lactate diagnostic protocols obtained by bicycle ergometry between 2015 and 2017 were examined. After outlier reduction to reduce the influence of extreme values on linear regression analysis, the number of data points was reduced from 1005 to 1010 (male and female), as described in the result section. The analysis of HRR and the weight-related performance at the IAT showed a significant correlation with a moderate coefficient of determination (R^2^ = 0.250). Thus, a prediction as well as a conclusion on physical performance based singularly on HRR decrease is not recommended, which supports the findings of other studies [19,20,21,22]. Daanen et al. conducted a systematic review in 2012 with five cross-sectional studies and eight longitudinal studies [15]. They also concluded that HRR is a significant parameter for training control but also dependent on many other factors [15]. The difference to this study was a small study population (10 to 95 subjects) in all previous studies [15]. In addition, in this retrospective examination of HRR a parameter transformation into separate HRR deltas was performed. By considering only the additional heart rate recovery after the prior HRR values (reducing the inherent correlation of subsequent HRR measurements), this results in all time-ranges being highly significant, and the overall adjusted coefficient of determination increasing to R^2^ = 0.250. Haraldsdottir et al. described pre- and post-season differences in relative and absolute HRR in female rowers [18]; a separate analysis of HRR in different timespans was not performed. Pre-seasonal athletes showed a faster drop in HRR in comparison to post-season values. These changes, however, showed no significant correlation with the VO2_max_ and they concluded that this parameter cannot be recommended for training control in this population [18]. The conclusions drawn by Haraldsdottir et al. are thus in line with the conclusions of our study. Schneider et al. examined the relevance of heart rate measurement and recovery [17] with a primary focus on team sports. They recommended heart rate monitoring for stress-related heart rates (HRmax and heart rate variability) in contrast to resting heart rates. Furthermore, training contexts, exhaustion level and the sport-specific performance should be considered for training control, in addition to vital parameters and HRR [18]. As this study examined HRR and performance level independent from sport-specific performance, future studies on HRR therefore need to consider these parameters and should distinguish different sporting activity types.

The gradient of the multiple linear regression and the coefficient of determination was higher for men in comparison to women in this retrospective analysis. This may be a result of the inhomogeneous clientele, including preventive medical check-up examinations and high-performance athletes, whilst women in a non-trained condition are less able to reach individual exhaustion.

### 4.2. Heart Rate Recovery in Preventative Medicine

However, HRR may not be a singular predictor for physical fitness, it should be examined in a greater context, as Nerderend et al. described a genetic dependence of HRR and vagal activity in a study with 491 twins and siblings in 2016 [23]. A consideration of submaximal heart frequencies and HRR of family members of the first degree also appear to be a possible approach for training control in preventative medicine [23] and should be taken into account in future work. Cole et al. showed a significant correlation of HRR and mortality in a longitudinal observation study with 2428 subjects [24]. A lack of heart rate recovery of less than 12 bpm was associated with an increased mortality; they attributed this to the proven relationship of higher vagal activity to mortality [25]. This study does show a comparable number of subjects, as studies for HRR and performance level in athletes included a small number of subjects [15,18], which results in a challenging interpretation. Dhoble et al. also found this connection in a retrospective analysis with 9030 volunteers in 2014 [26]. In addition, changes in heart rate recovery can be a sign for autonomic dysfunction and should lead to further examination of the patient to avoid adverse cardiac events [27]. Future studies should include HRR as a longitudinal parameter in athletes and patients.

In conclusion, HRR is not a useful single parameter for training control in healthy individuals [15,18]. However, HRR is a relevant parameter for longitudinal health monitoring and mortality, especially for individuals with cardiac pathologies and to predict metabolic syndrome [28,29,30,31]. It seems sensible to include HRR as a further parameter for individual training control, but not to be considered singularly in terms of performance [15,18]. In order to use HRR, in the outpatient and inpatient sector, a standardized measurement protocol after defined recovery times is necessary in order to draw adequate comparisons and conclusions for the respective recommendations in preventative medicine.

### 4.3. Limitations

Due to the retrospective analysis and the manual entry of the measured values during lactate performance diagnostics, incorrect entries must be considered. However, these were minimized in advance by means of a plausibility check of the entire data set. Furthermore, it needed to be considered that HR changes and also HRR may differ between different sports, as a difference between cycle ergometry and a 6-min walk test were observed [32,33,34]. At the same time, ergometry offers a simple and inexpensive measuring method that can be performed in outpatient and inpatient settings and represents an adequate procedure for popular sports and preventive medicine.

## 5. Conclusions

In this study, HRR and the weight-related performance at the IAT showed a significant correlation with a mean strength. Thus, a prediction as well as a conclusion on physical performance based singularly on HRR decrease is not recommended. However, in preventative medicine, HRR should be measured and observed longitudinally for analysis of vagal activity to draw inferences of mortality. A regular measurement, observation and evaluation of the HRR in annual health check-up examinations is recommended. Furthermore, it is necessary to establish resource-efficient diagnostics in future studies to predict essential parameters for training management without measuring blood lactate for preventative medicine.

## Figures and Tables

**Table 1:** Baseline characteristics of male and female subjects.

		Male (n = 765)		Female (n = 469)	
		Mean ± SD		Mean ± SD	
Age (years)		46.61	±18.60	48.24	±19.36
Height (cm)		180.33	±7.93	166.25	±7.79
Weight (kg)		84.77	±13.53	67.12	±12.37
Pmax/kg (W/kg)		2.78	±1.02	2.16	±0.91
P_mean_ (W)	Mean power	141.19	±48.68	89.85	±35.44
HR_min_	Minimum HR	70.38	±12.41	73.30	±12.53
HR_mean_	Mean HR	123.32	±15.94	124.00	±16.83
HR_max_	Maximum HR	170.13	±20.78	166.99	±20.93
HR_pw1_	HR 1 min post workout	144.85	±20.04	142.43	±22.63
HR_pw3_	HR 3 min post workout	119.26	±17.44	115.66	±20.04
HR_pw5_	HR 5 min post workout	108.59	±16.60	103.80	±18.57
HRR	HR_max_ − HR_5min_	61.54	±15.02	63.19	±14.04
W/kg (IAT) (W/kg)		1.98	±0.77	1.58	±0.64
HR (IAT)		141.12	±19.46	143.99	±18.66

**Table 2:** Multiple linear regressions of weight-related performance at IAT in W/kg using gender (m = 0, f = 1) and HRR values after 1, 3, and 5 min.

	coeff.	SE	*p* Value	coeff.	SE	*p* Value	coeff.	SE	*p* Value
bias	0.4755	0.1125	<0.0001	0.4440	0.1111	0.0001	0.5221	0.1084	<0.0001
gender	−0.3256	0.0357	<0.0001	−0.3231	0.0357	<0.0001	−0.3260	0.0359	<0.0001
HRR_1min_	0.0054	0.0032	0.0961						
HRR_3min_	−0.0113	0.0033	0.0007	−0.0088	0.0030	0.0030			
HRR_5min_	−0.0159	0.0027	<0.0001	−0.0163	0.0026	<0.0001	−0.0223	0.0017	<0.0001
R^2^ (adj.)	0.219			0.217			0.210		
SE (W/kg)	0.519			0.512			0.522		

**Table 3:** Multiple linear regressions of weight-related performance at IAT in W/kg using gender (m = 0, f = 1) and HRR deltas.

	coeff.	SE	*p* Value
bias	0.5154	0.1114	<0.0001
gender	−0.3256	0.0357	<0.0001
HRR_1min_	−0.0232	0.0028	<0.0001
HRR_3min_ − HRR_1min_	−0.0262	0.0023	<0.0001
HRR_5min_ − HRR_3min_	−0.0150	0.0026	<0.0001
R^2^ (adj.)	0.250		
SE (W/kg)	0.520		

## Data Availability

The data presented in this study are available on request from the corresponding author. The data are not publicly available due to data privacy regulations.
